# Marked increase in cryptosporidiosis cases, Spain, 2023

**DOI:** 10.2807/1560-7917.ES.2024.29.28.2300733

**Published:** 2024-07-11

**Authors:** Marina Peñuelas Martinez, David Carmena, Bernardo R Guzmán Herrador, Margarita Palau Miguel, Gabriela Saravia Campelli, Rosa María García Álvarez, María Guerrero-Vadillo, Alejandro Dashti, Pamela C Köster, Esperanza Guevara Alemany, Fernando Simón Soria, Isabel Fuentes Corripio, Carmen Varela Martínez, María José Sierra Moros, working group for the National Surveillance Network, Collaborators, Nicola Lorusso, Isabel M Vázquez Rincón, Alejandra Pérez Pérez, Cristina Navarro Gistau, An L D Boone, Sara Iglesias Martinez, Teresa González Cortijo, Marta Ramirez Cases, Laura García Hernández, Marta Pacheco Gorostiaga, Luis Javier Viloria Raymundo, Mª del Henar Marcos Rodríguez, Virginia Álvarez Río, Conchita Izquierdo Gómez, Aurora Sabrià Sunyé, Ninoska López Berrios, Violeta Ramos Marín, Enrique Mansilla Ferrer, Cristina Vicedo García, Ignacio Pérez Sánchez, Noa Batalla Rebollo, Andrés Aragón Peña, Manuel José Velasco Rodriguez, Daniel Castrillejo Pérez, Maria Dolores Chirlaque López, Alonso Sánchez-Migallón Naranjo, Jesús Castilla, Itziar Casado, Fernando González Carril, Adrían Hugo Aguinagalde, Ana Carmen Ibáñez Pérez

**Affiliations:** 1 Escuela Internacional de Doctorado, Universidad Nacional de Educación a Distancia (UNED), Madrid, Spain; 2 National Centre of Epidemiology, Instituto de Salud Carlos III, Madrid, Spain; 3 CIBER in Epidemiology and Public Health (CIBERESP), Madrid, Spain; 4 National Centre of Microbiology, Instituto de Salud Carlos III, Majadahonda, Spain; 5 CIBER in Infectious Diseases (CIBERINFEC), Madrid, Spain; 6 Coordinating Centre for Health Alerts and Emergencies, Ministry of Health, Madrid, Spain; 7 General Subdirectorate of Environmental Health, Ministry of Health, Madrid, Spain; 8 Hospital Clínico Universitario de Santiago (A Coruña), Santiago de Compostela, Spain; 9 Faculty of Health Sciences, Alfonso X El Sabio University (UAX), Villanueva de la Cañada, Madrid, Spain; 10 Faculty of Medicine, Alfonso X El Sabio University (UAX), Villanueva de la Cañada, Spain; 11 The members of the working group for the National Surveillance Network are listed under Collaborators; * These authors contributed equally to the work and share first authorship.; ** These authors contributed equally to the work and share last authorship.

**Keywords:** waterborne infection, surveillance, *Cryptosporidium*, outbreak, increase, Spain

## Abstract

**Background:**

By mid-September 2023, several event notifications related to cryptosporidiosis had been identified from different regions in Spain. Therefore, a request for urgent notification of cryptosporidiosis cases to the National Surveillance Network was launched.

**Aim:**

We aimed at assessing the extent of the increase in cases, the epidemiological characteristics and the transmission modes and compared to previous years.

**Methods:**

We analysed data on case notifications, outbreak reports and genotypes focusing on June–October 2023 and compared the results to 2016–2022.

**Results:**

In 2023, 4,061 cryptosporidiosis cases were notified in Spain, which is an increase compared to 2016–2022. The cumulative incidence was 8.3 cases per 100,000 inhabitants in 2023, sixfold higher than the median of 1.4 cases per 100,000 inhabitants 2016–2022. Almost 80% of the cases were notified between June and October. The largest outbreaks were related to contaminated drinking water or swimming pools. *Cryptosporidium hominis* was the most common species in the characterised samples (115/122), and the *C. hominis* IfA12G1R5 subtype, previously unusual in Spain, was detected from 76 (62.3%) of the 122 characterised samples.

**Conclusions:**

A substantial increase in cryptosporidiosis cases was observed in 2023. Strengthening surveillance of *Cryptosporidium* is essential for prevention of cases, to better understand trends and subtypes circulating and the impact of adverse meteorological events.

**Key public health message**

**What did you want to address in this study and why?**

*Cryptosporidium* is a parasite causing a gastrointestinal illness called cryptosporidiosis affecting people and animals. In 2023, a substantial increase in human cryptosporidiosis cases was observed in Spain. In this study, we investigated the extent of the increase and distribution of cases and the sources of the largest outbreaks.

**What have we learnt from this study?**

The increase was seen throughout Spain, and the incidence per 100,000 population was sixfold higher than the median of 2016–2022. Almost 80% of the 4,061 cases occurred during summer. The largest outbreaks were related to consumption of contaminated drinking water or to the use of swimming pools. An unusual subtype of *Cryptosporidium hominis* was the most common among characterised samples.

**What are the implications of your findings for public health?**

Continuous surveillance is crucial in identifying increases in cases and changes in the pattern of the disease in Spain and elsewhere. Surveillance of cryptosporidiosis should be conducted under the One Health approach with epidemiological, microbiological and environmental data analysed and interpreted together.

## Introduction

Cryptosporidiosis is a gastrointestinal disease occurring worldwide and caused by protozoa of the *Cryptosporidium* genus. Infections in humans are most often caused by *Cryptosporidium hominis,* with humans as the main reservoir, or by zoonotic *Cryptosporidium parvum,* usually found in the intestine of livestock. The pathogen is transmitted via the faecal-oral route, either directly from humans or animals or indirectly from ingestion of contaminated water or food [1]. The infective dose is low, ingestion of 10–30 oocysts can cause an infection [2]. Reported large outbreaks have been mainly associated with faecal contamination of drinking water and recreational water, such as swimming pools, or to a lesser extent, unprocessed food, contact with animals, and occasionally, person-to-person transmission in specific settings such as daycare centres [3,4]. The oocysts, the transmissive stage of the parasite, are highly resistant to disinfection. Ozonation and ultraviolet radiation inactivate *Cryptosporidium* oocysts in drinking water [5,6].

According to the annual national report of 2021, the notification rate of cryptosporidiosis in Spain (1.0 cases/100,000 inhabitants) was below the notification rate in the European Union (EU) (1.8 cases/100,000 inhabitants) [7]. Ireland and Luxembourg had higher notification rates (> 10 cases/100,000 inhabitants) [1]. The EU notification rate in 2021 was lower than before the COVID-19 pandemic [1,7]. The age distribution, with the highest rates among children < 4 years, and a bimodal seasonal distribution, with one peak in early spring and another in late summer–early autumn, were similar to previous seasons [7]. Between 2010 and 2011, Sweden recorded the two largest outbreaks reported in Europe to date with around 47,000 people diseased after consumption of contaminated drinking water [8].

Between 2016 and 2020, a median of 569 laboratory-confirmed cases were annually notified to the National Surveillance Network of Spain [9]. The numbers ranged from 137 cases in 2020 to 1,582 in 2018. During these years, a median of three outbreaks were annually reported, from no outbreaks in 2020 to seven in 2017. Nine outbreaks involved only two individuals and were restricted to the same household. The largest outbreak, with an unknown source of infection, affected 13 individuals in 2018. Previous increases in incidence were seen in some regions of Spain before cryptosporidiosis became notifiable [10]. In Spanish studies covering a larger number of characterised cases between 2007 and 2017, most human cases were caused by subtype family (hereinafter called family) Ib of *C. hominis* (69.2%) or by family IIa of *C. parvum* (21.0%) [11-16]. These results were in line with 2015–2020 data from the Parasitology Reference and Research Laboratory (LRIP) of the National Center for Microbiology where *C. hominis* (81.5%; 233/286) dominated over *C. parvum* (16.8%; 48/286), *C. meleagridis* (1.4%; 4/286) and *C. cuniculus* (0.3%; 1/286). Most common families were Ib of *C. hominis* (67.1%; 156/233) and IIa of *C. parvum* (4/48). Subtype IbA10G2 accounted for most infections. In other European countries, *C. parvum* has been more common than *C. hominis,* such as in the United Kingdom (UK) (58% vs 43%) [17] and France (72% vs 24%) [18]. The *C. hominis* subtype IbA10G2 was also dominant in sporadic cases and outbreaks in different countries between 2000 and 2020 [19,20].

According to the current Spanish legislation, *Cryptosporidium* species (spp.) are not systematically monitored in surface water, groundwater or in swimming pools. In outbreak investigations, public health authorities can request sampling of water. In drinking water, other microorganisms are normally included in the monitoring as possible indicators of pathogens. Under certain circumstances, such as increased water turbidity, public health authorities may require testing for *Cryptosporidium* [21,22].

On 15 September 2023, following reports of several events related to cryptosporidiosis in different regions of Spain, the Coordinating Centre for Health Alerts and Emergencies (CCAES) of the Ministry of Health, under the framework of the National Early Warning and Rapid Response System, requested the focal points in the regions urgently notify cryptosporidiosis cases and clusters through the National Surveillance Network, to assess the epidemiological situation. In this article, we present the investigation of cryptosporidiosis epidemiology in Spain in 2023.

## Methods

### Surveillance of human cases and case definition

Cryptosporidiosis became a notifiable disease in Spain in 2015 [23]. Seventeen of the 19 Spanish autonomous communities and autonomous cities (regions) report case-based data on laboratory-confirmed cases to the National Centre of Epidemiology (CNE) using an electronic platform (SiVIEs). One region started surveillance in 2020. The case definition according to the National Surveillance Network [24] is based on the European definition of confirmed cases, which includes cases that fulfil clinical criteria and are laboratory-confirmed [25]. Cases without information on clinical symptoms are included and considered as confirmed if they have a positive laboratory result. An outbreak is defined as two or more individuals with clinical symptoms compatible with cryptosporidiosis, regardless of laboratory confirmation but with an epidemiological link to a laboratory-confirmed case or to a source in which *Cryptosporidium* spp. has been confirmed. Outbreaks need to be reported to the outbreak reporting system. For each outbreak, an ad-hoc definition of confirmed, probable and suspected cases is established when necessary for the epidemiological investigation. Laboratory-confirmed cases in a region at a given time without a clear epidemiological link are defined as a cluster and are not reported as outbreaks. The following information is included in case notifications: time (onset of symptoms, date of diagnosis and reporting), place (residence, exposure and reporting) and person (age, sex, hospitalisation and death) as well as whether the case is a single case or outbreak-related. In outbreak reports, the suspected mode of transmission, the outbreak setting, the vehicle, contributory factors and the implemented control measures are included. According to the National Surveillance Network, a case is considered hospitalised if at least one night is spent in a hospital.

Local and regional public health authorities are responsible for outbreak detection, investigation and control and should submit outbreak reports once the investigation is completed. In case of multi-regional outbreaks, urgent notification to the CNE and the CCAES is required.

### Study population

We included data on all laboratory-confirmed cases with symptom onset between January and December 2023 and notified to the National Surveillance Network by 4 April 2024. In the absence of the date of onset, the earliest available date was used: either date of diagnosis or, if missing, date of notification. We also analysed the information included in the outbreak reporting system. In addition to the information included in the outbreak reports, we added other details or narrative information on potential sources linked to the outbreak or to the cluster after direct consultations with the regional focal points.

### Comparison of 2023 data to 2016–2022

To calculate the cumulative incidences, the numerator was the total number of cases notified each year (excluding imported cases and non-resident cases) and the denominator was the population resident in Spain on 1 January of the corresponding year, according to the National Institute of Statistics [26]. The population of the regions with no notified cases in a certain year were excluded from the denominator of that year. For 2023, the same population as in 2022 was assumed as this information was not yet available.

### Laboratory analyses

Testing for *Cryptosporidium* from clinical samples from humans is conducted at hospital clinical microbiology laboratories. The testing criteria are not harmonised. In recent years, there has been a significant improvement in the diagnostic capacity of the local laboratories, since techniques, such as rapid immunochromatography or molecular techniques based mainly on real-time PCR, have been incorporated as the first-line diagnostic methods complementing, or even replacing, the traditional diagnosis by conventional microscopy. The LRIP receives clinical samples on a voluntary basis and conducts diagnostic testing and molecular genotyping on request. Thus, the LRIP cannot assess the geographical or temporal representativeness at the national level. We subtyped samples using molecular (PCR and Sanger sequencing) methods. At LRIP, detection and identification of *Cryptosporidium* species was done by a PCR targeting a partial fragment of the gene codifying the small subunit (*ssu*) rRNA [27]. Samples with a positive result by (*ssu*)-PCR were subsequently reassessed by a PCR targeting a partial fragment of the 60 kDa glycoprotein gene (*gp60*) to ascertain *Cryptosporidium* families and subtypes [28].

## Results

**Figure 1:**
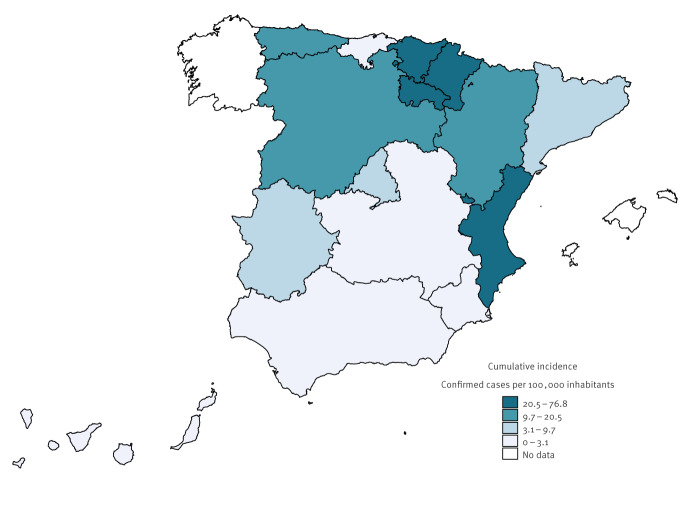
Cumulative incidence of notified cryptosporidiosis cases, by region, Spain, 2023

A total of 4,061 laboratory-confirmed cases of cryptosporidiosis were notified to the National Surveillance Network between January and December 2023. Fourteen cases were categorised as imported. The incidence was 8.3 cases per 100,000 inhabitants, a substantial increase from the years between 2016 and 2022 with a median incidence of 1.4 cases per 100,000 inhabitants (minimum: 0.3/100,000 in 2020 and maximum: 3.8/100,000 in 2018) [9]. Figure 1 shows the cumulative incidence in 2023 by region. The median number of notified cases per region between 2016 and 2022 varied between 0 and 235 (minimum of 0 and maximum of 668 notified by a single region in 2018). In 2023, in all regions, except for two regions that had never reported cryptosporidiosis, incidences were higher than the median for 2016–2022, according to previously published national reports on cryptosporidiosis [9]. The increase in the number of notified cases in 2023 compared to 2016–2022 varied between regions but was > 60% in all regions notifying cases. The smallest increases were observed in two regions reporting the highest number of cases in 2016–2022.

### Seasonality

**Figure 2:**
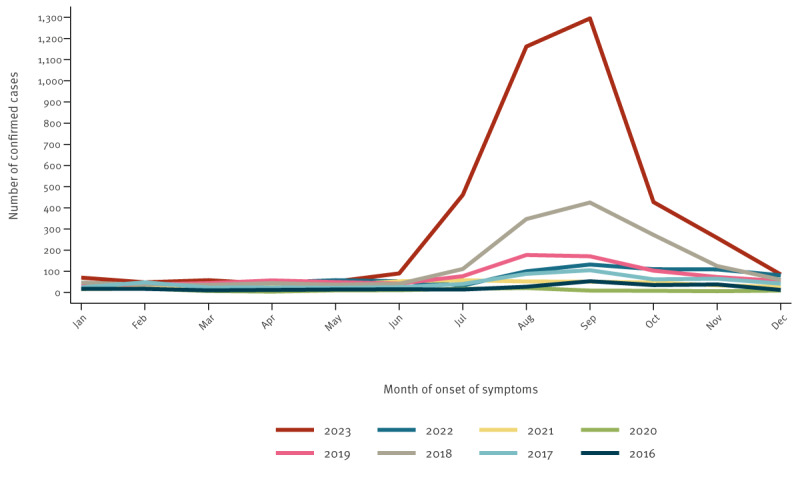
Notified cryptosporidiosis cases, by month, Spain, 2016–2023 (n = 8,792)

In 2023, most domestic cases were reported in September (n = 1,295), August (n = 1,162) and July (n = 461) (Figure 2). In June, 90 cases were notified, and in October, 427 cases. Thus, 84.9% (3,435/4,047) of the domestic cases were notified between June and October.

### Age and sex distribution

**Figure 3:**
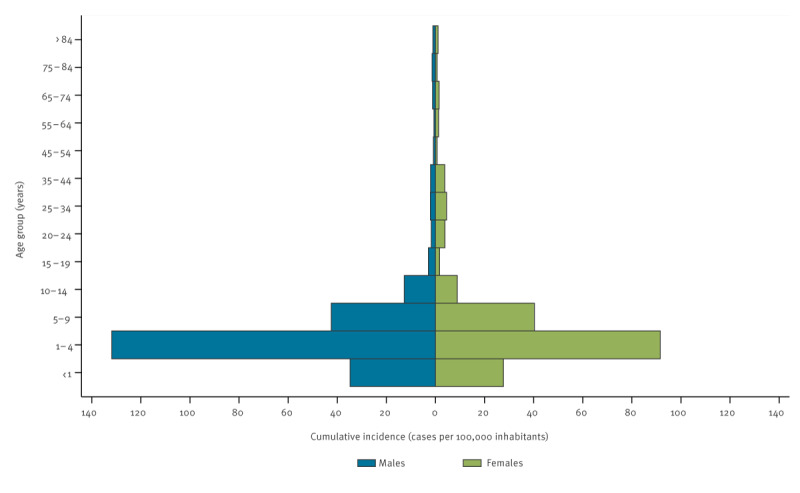
Cumulative incidence of notified cases of cryptosporidiosis, by age group and sex, Spain, June–October 2023

Between June and October, the highest cumulative incidences were recorded in children aged 1–4 years (131.9/100,000 in males and 91.6/100,000 in females), followed by children aged 5–9 years (42.5/100,000 in males and 40.3/100,000 in females), in both sexes (Figure 3). Before June and after October, the most affected age group was children aged 1–4 years (7.8/100,000 in males and 4.9/100,000 in females before June and 16.1/100,000 in males and 14.2/100,000 in females after October), but the second most affected age group was those aged < 1 year (6.3/100,000 in males and 2.0/100,000 in females before June and 6.3/100,000 in males and 3.4/100,000 in females after October).

### Hospitalisation and deaths

**Figure 4:**
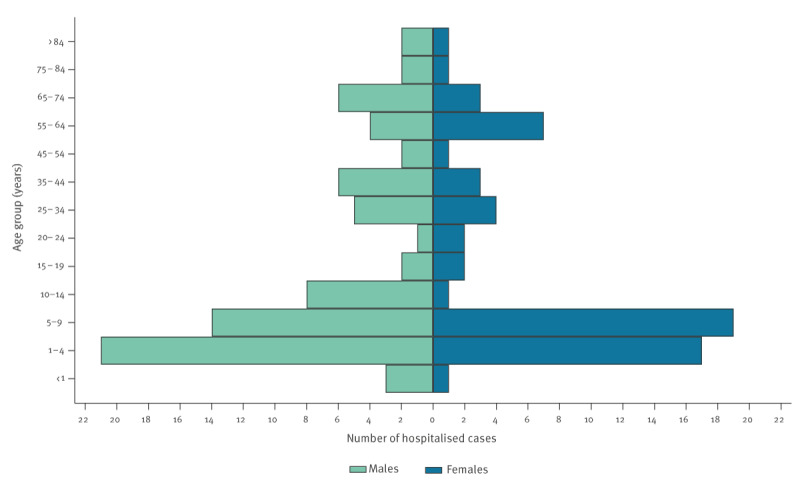
Hospitalised cases of cryptosporidiosis, Spain, June–October 2023 (n = 138)

In 2023, information on hospitalisation was available for 2,460 (60.6%) cases; a total of 170 cases were hospitalised (6.9% of those with the information available) (Figure 4). Between June and October, 138 cases were hospitalised. The median age of hospitalised cases was 8 years (interquartile range (IQR): 37, range: 0–93 years), 9.5 years (IQR: 39) for males and 8 years (IQR: 32) for females. The age groups most often hospitalised between June and October were children aged 1–4 and 5–9 years, with 38 and 33 cases in each group, respectively, representing less than 10% of the cases in these groups. No cryptosporidiosis associated deaths were reported in 2023.

### Outbreaks

Although information on a possible link to an outbreak was missing for 2,559 (63.0%) of the confirmed cases, at least 355 confirmed cases were linked to 71 outbreaks. Only four outbreaks, with two cases each, were reported before June or after October, according to the information in the outbreak reporting system. Four outbreaks had more than 50 cases, including also cases that were not laboratory-confirmed. An outbreak in June 2023 with 53 cases was linked to a misuse of an urban fountain for recreational purposes, another outbreak in August with 119 cases was linked to a swimming pool. In September, 443 cases in an educational institution for adults acquired the infection most likely via water, although *Cryptosporidium* spp. were not detected from water samples (Table 1). In another outbreak in September, 539 cases were associated with consumption of contaminated drinking water from a water supply network. Table 1 presents the main characteristics of the outbreaks with more than five cases. In the remaining outbreaks (n = 51), accounting for 71.8% of the outbreaks, two cases (n = 32), three cases (n = 10), four cases (n = 4) or five cases (n = 5) were notified. These outbreaks were linked to household transmission (n = 23), private swimming pools (n = 16), hotels (n = 6) and nurseries or schools (n = 5). In one outbreak, the most likely setting could not be identified.

**Table 1:** Main characteristics of cryptosporidiosis outbreaks with more than five cases, Spain, June–October 2023 (n = 20)^a^

ID	Setting^b^	All cases^c^	Confirmed cases (n)				Comments
			Total	Aged < 15 years	Males	Females	
1	Community	539	69	28	34	35	*Cryptosporidium hominis* detected in water supply, different genotypes from case samples
2	Closed setting	443	5	0	3	2	Water tank most likely source, *Cryptosporidium* not detected from water samples
3	Swimming pool	119	27	19	11	16	Public pool
4	Urban fountain	53	53	53	33	20	Not drinking water
5	Swimming pool	15	15	15	7	8	Public pool
6	Hotel	14	1	0	Unknown		Identified after the hotel closed at the end of the season
7	Swimming pool	13	13	13	7	6	School pool
8	Community	11	11	9	6	5	*Cryptosporidium* spp. detected in water supply, different genotypes involved (human samples)
9	Swimming pool	10	10	8	6	4	Public pool
10	Swimming pool	10	10	10	4	6	Public pool
11	Swimming pool	10	5	5	3	2	Camping pool
12	Swimming pool	8	8	6	5	3	Public pool
13	Hotel^d^	8	Unknown	NA	NA		No comments
14	Household	8	8	8	4	4	No comments
15	Swimming pool	7	7	7	5	2	Public pool
16	Swimming pool	7	7	7	4	3	No comments
17	Hotel^d^	7	Unknown	NA	NA		No comments
18	Nursery	7	7	6	6	1	No comments
19	Community	6	6	3	4	2	No comments
20	Hotel^d^	6	Unknown	NA	NA		No comments

ID: identification code; NA: not applicable; spp.: multiple species.

^a^ Notified to the National Centre of Epidemiology case-based and the outbreak-based reporting system.

^b^ Most likely setting according to local epidemiological investigations.

^c^ Outbreaks with a missing number of confirmed cases are those for which no cases related to the outbreak have been identified or reported in the case-based reporting system.

^d^ Hotel-associated outbreaks among foreign tourists; the source of the infection was not identified.

As shown in Table 1, most of these outbreaks were related to exposure in swimming pools. Three regions reported three outbreaks associated with consumption of drinking water. In two outbreaks, public health authorities declared the water supply unsafe after detecting the parasite from water samples, although the number of oocysts was low (< 5/100 L). The sediments of six water samples from one of these outbreaks were available for molecular testing. *Cryptosporidium hominis* was detected from one of them but attempts to determine the family and subtype failed repeatedly. Molecular genotyping was not done from any of the water samples, probably due to the low numbers of oocysts obtained. Lack of genotyping data precluded the confirmation of the waterborne origin of the outbreak, although this was the most likely explanation. After appropriate treatment of the water supply and not detecting any oocysts from the water samples, no further cases were notified.

### Molecular investigation of cases identified in 2023

In 2023, the LRIP observed an unusual increase in the number of requests from the regions. A median of 127 samples per year were analysed 2016–2022 compared to 365 in 2023. In 2023, seven outbreaks and clusters, between September and October, from five different regions were investigated (Table 2). *Cryptosporidium hominis* was isolated from 94.3% (115/122) of the case samples and *C. parvum* from 5.7% (7/122). No other *Cryptosporidium* species were identified. *Cryptosporidium hominis* was detected in all clusters investigated, whereas *C. parvum* was detected in only two regions. In clusters with more than 10 samples investigated, 1–2 *Cryptosporidium* species and 3–4 different genotypic families were identified. The *C. hominis* IfA12G1R5 subtype (family If) was identified from 62.3% (76/122) of case samples, regardless of geographical origin (Table 2). The *C. hominis* IbA10G2 subtype (family Ib), the predominant genetic variant in Spain to date, was identified from only one case.

**Table 2:** Diversity and frequency of *gp60* families of *Cryptosporidium hominis* and *parvum* from case samples from cryptosporidiosis outbreaks and clusters analysed at the LRIP, Spain, June–October 2023 (n = 122)

Outbreaks/ Clusters	*Cryptosporidium hominis*					*Cryptosporidium parvum*			Total
	NA	Ia	Ib	Id	If	NA	IIa	IId	
A	4	1	1	1	39	1	4	1	52
B (outbreak 8^a^)	0	0	0	1	3	0	0	0	4
C	11	3	2	1	16	0	0	0	33
D (outbreak 1^a^)	3	6	3	0	11	1	0	0	24
E (outbreak 2^a^)	2	0	0	0	3	0	0	0	5
F	0	0	0	0	2	0	0	0	2
G	0	0	0	0	2	0	0	0	2
Total (n)	20	10	6	3	76	2	4	1	122
Percentage	16.4	8.2	4.9	2.5	62.3	1.6	3.3	0.8	100

LRIP: Parasitology Reference and Research Laboratory of the National Center for Microbiology; NA: not available.

^a^ The number refers to the outbreak identification code in Table 1.

## Discussion

In 2023, a sixfold increase of cryptosporidiosis cases was observed in Spain compared to the median of previous years. The national public health authorities launched a request for a rapid update and notification of cases and outbreaks following reports of multiple events involving notifications of cryptosporidiosis cases in several regions. It was then when the considerable national increase became evident.

Information extracted from the outbreak reports together with additional information from consultations with the regions revealed two different profiles. Firstly, a large proportion of cases were related to exposure to swimming pools and other recreational waters. Secondly, some outbreaks, a few with a large magnitude, were related to consumption of contaminated drinking water from the water supply network.

This increase could be due to a combination of factors. According to the Spanish Meteorological Agency (AEMET) [29], the summer of 2023 was exceptionally hot and wet. Apart from the Canary Islands, which experienced two heat waves in August, the rest of Spain experienced four heat waves, two in July and two in August, thus the summer became the third hottest of the 21st century. Maximum rainfalls were observed in June, preceded by the driest spring of this century, and the summer was the third wettest. The extreme heat could also have led to a larger use of swimming pools and recreational waters and, therefore, a greater crowding in these facilities. Also, as previously described in the literature [30,31], adverse meteorological events could affect drinking water treatment plants. Another factor to consider is that in recent years there has been a significant improvement in diagnostic capacity at local laboratories, which may have influenced the increase in case detection. This fact alone does not explain the accumulation of notified cases, but the diagnostic capacity should be further mapped. Also, the enhanced surveillance conducted since September 2023 under the framework of the National Early Warning and Rapid Response System, may have increased awareness among those notifying. Additional sources of infection and transmission pathways, such as the consumption of fresh produce cannot be ruled out.

An increase in cryptosporidiosis cases was also observed in other European countries. In October 2023, the European Centre for Disease Prevention and Control (ECDC) reported an increase in cryptosporidiosis cases in Ireland, Luxembourg, the Netherlands and the UK from the end of August and particularly in September 2023 [32]. The ECDC pointed out a combination of contributing factors related to travelling abroad and extreme weather conditions such as heat waves, heavy rains and floods. A recent UK publication links this to recreational water bathing in the UK or abroad, including the use of swimming pools and travel to various destinations, e.g. Spain [33]. Ireland and the UK informed the CCAES about an increase in cases with a history of travel to Spain during the summer, cases mainly linked to resorts, hotels and campsites in the Mediterranean area. This increase was probably due to environmental conditions favouring transmission of *Cryptosporidium* in southern Europe and summer travel patterns towards these countries [32].

Our molecular analyses indicate that the infections were mainly caused by unusual genetic variants (subtype IfA12G1R5, and to a much lesser extent IbA12G3 and IbA13G3), suggesting the replacement of common variants from previous years (primarily subtype IbA10G2). Transmission of *C. hominis* decreased in England and Wales [34,35] and New Zealand [36] during the COVID-19 pandemic. A recent study from the United States (US) [37] showed that the traditionally predominant *C. hominis* subtype IbA10G2 had largely disappeared and been in recent years replaced by IfA12G1R5. Authors in the US study suggest that multiple introductions and genetic recombination events, with a subsequent adaptive selection, could have led to the emergence of this hyper-transmissible subtype, which could be consistent with the findings of other countries. In addition to Spain, IfA12G1R5 has also been identified in other European countries, such as in Denmark, Germany, Ireland, the Netherlands, Sweden and the UK [33,38] and has become common in Australia and New Zealand [39,40].

The number of cases notified to the National Surveillance network clearly underestimate the true number of cases due to (i) asymptomatic or mild cases not seeking medical care and, if they do, laboratory testing is not usually performed and (ii) under-reporting of confirmed cases and outbreaks in some regions. In addition, reporting is not done in real time, but depends on the regional authorities checking and completing the information, with significant differences between them, which may cause delays in the detection of increases in the incidence. Information on the source of exposure should be included in case notifications. However, it is difficult to verify the exposure for a sporadic case, especially if there is a lack of resources to interview single cases.

Laboratory capacity and testing criteria may also differ between regions and hospitals. We cannot exclude the possibility of variations in the sampling criteria between regions and settings, with paediatricians more often seeking a laboratory diagnosis and with some regions trying to confirm all cases suspected to be part of an outbreak while others do not. In addition, the choice of laboratory methods applied may affect both the number and the proportion of cases detected, since traditional diagnosis (primarily microscopical techniques) usually requires a specific request for a stool parasites test, whereas analysis for *Cryptosporidium* spp. is included in multi-target PCR panels for diagnosis of enteric pathogens, even if the parasite is not suspected. Unfortunately, in most cases there is no information on the analytical method. The subtyping results from the LRIP do not give information on prevalence, as the samples are sent on a voluntary basis and depend on the interest of those sending samples. As low numbers of oocysts were obtained from the water samples, we could not perform genotyping. Thus, it was difficult to confirm water contamination as the source of the outbreaks linked to consumption of drinking water.

The number of notifications decreased during the autumn and winter months, probably due to weather conditions and reduced use of swimming pools and recreational waters, but a delay in reporting cannot be excluded. However, monitoring is essential ahead of the 2024 summer as possible heat waves, extreme rainfalls and other adverse weather events may increase the risk of waterborne diseases [41,42], including cryptosporidiosis. The capacity of water treatment plants to cope with these extreme events may determine a risk of waterborne disease outbreaks, which may be higher in small urban or rural areas, especially if disinfection is based only on chlorine derivates, which do not eliminate oocysts [6]. Local authorities should continue preparing water safety plans to identify and mitigate risks through effective and appropriate preventive measures. The Ministry of Health has developed, together with the Spanish Association of Water Supply and Sanitation, a tool to facilitate the preparation of water safety plans and since March 2023, a practical guide is available.

Surveillance of cryptosporidiosis should be conducted under the One Health approach with epidemiological, microbiological and environmental data analysed and interpreted together. Protocols and indications for monitoring *Cryptosporidium* in water should be reviewed to determine when and where, either temporally or spatially, this parasite should be routinely monitored. Determining the role of adverse meteorological events in waterborne infections is a key public health issue. Research combining epidemiological, microbiological and meteorological data should be encouraged to analyse associations between precipitation or extreme temperatures and waterborne infections and establish models for prevention. Such studies should explore potential effect modifiers, such as specific types of microorganism, geographic region, season, type of water supply or water treatment, to evaluate the relationship between adverse weather effects and waterborne infections and identify areas where action is needed to minimise the negative impact of climate change on health. To prevent outbreaks related to exposure to swimming pools and recreational waters, the correct maintenance of swimming areas will be crucial. Information campaigns on how to minimise the risk of contracting cryptosporidiosis, such as on compliance to hygiene recommendations before entering swimming pools and recreational waters. This information should be made available to families with young children, especially those visiting public swimming pools or other water recreation sites, zoos or farms, as well as participants in mass sporting events involving outdoor swimming.

## Conclusions

Given this recent increase, it is important to strengthen surveillance and molecular analysis to better understand trends and subtypes circulating in our country and elsewhere in Europe. In outbreak investigations, a comprehensive epidemiological and microbiological investigation, including the collection and analysis of samples from patients and suspected source must be carried out to determine the magnitude of the outbreak, the source of infection and contributing factors to implement the appropriate corrective measures. Notification of outbreaks must be accompanied with epidemiological, environmental and microbiological information. It is also essential to design environmental surveillance protocols for sampling and analysis of *Cryptosporidium* spp. from environmental samples.

## Affiliations of the collaborators

All affiliations are in Spain. Andalucía: Nicola Lorusso and Isabel M Vázquez Rincón, General Directorate of Public Health and Pharmaceutical Regulation, Regional Ministry of Health and Consumption, Andalusia; Aragón: Alejandra Pérez Pérez and Cristina Navarro Gistau, Subdirección de Salud Pública de Zaragoza, Gobierno de Aragón; Asturias: An L D Boone and Sara Iglesias Martinez, Dirección General de Salud Pública y Atención a la Salud Mental, Gobierno de Asturias, Oviedo; Balearic Islands: Teresa González Cortijo and Marta Ramirez Cases, Dirección General de Salud Pública; Canary Islands: Laura García Hernández, General Directorate of Public Health of the Canary Health Service; Cantabria: Marta Pacheco Gorostiaga and Luis Javier Viloria Raymundo, Dirección General de Salud Pública; Castilla y León: Mª del Henar Marcos Rodríguez and Virginia Álvarez Río, Dirección General de Salud Pública, Valladolid; Cataluña: Conchita Izquierdo Gómez and Aurora Sabrià Sunyé, Public Health Agency of Catalonia; Ceuta: Ninoska López Berrios and Violeta Ramos Marín, Consejería de Sanidad, y Servicios sociales, Ciudad Autónoma de Ceuta; Comunidad Valenciana: Enrique Mansilla Ferrer and Cristina Vicedo García, Subdirecció General d’Epidemiologia i Vigilància de la Salut; Extremadura: Ignacio Pérez Sánchez and Noa Batalla Rebollo, Dirección General de Salud Pública, Servicio Extremeño de Salud, Merida; Madrid: Andrés Aragón Peña and Manuel José Velasco Rodriguez, Dirección General de Salud Pública de la Comunidad de Madrid; Melilla: Daniel Castrillejo Pérez, Consejería de Políticas Sociales y Salud Pública; Murcia: Maria Dolores Chirlaque López and Alonso Sánchez-Migallón Naranjo, Regional Health Council, IMIB-Arrixaca, Murcia University - CIBER Epidemiologia y Salud Pública (CIBERESP); Navarra: Jesús Castilla and Itziar Casado, Instituto de Salud Pública de Navarra – Navarra Institute for Health Research, IdiSNA – CIBER Epidemiologia y Salud Pública, CIBERESP, Pamplona; País Vasco: Fernando González Carril and Adrían Hugo Aguinagalde, Departamento de Salud del País, Vasco; La Rioja: Ana Carmen Ibáñez Pérez, Dirección General de Salud Pública, Consumo y Cuidados de la Consejería de Salud de La Rioja, Logroño.
